# Uses of Mobile Device Digital Photography of Dermatologic Conditions in Primary Care

**DOI:** 10.2196/mhealth.8257

**Published:** 2017-11-08

**Authors:** Jennifer L Pecina, Kirk D Wyatt, Nneka I Comfere, Matthew E Bernard, Frederick North

**Affiliations:** ^1^ Department of Family Medicine, Mayo Clinic Rochester, MN United States; ^2^ Department of Pediatric and Adolescent Medicine, Mayo Clinic Rochester, MN United States; ^3^ Departments of Dermatology and Laboratory Medicine & Pathology, Mayo Clinic Rochester, MN United States; ^4^ Department of Primary Care Internal Medicine, Mayo Clinic Rochester, MN United States

**Keywords:** telemedicine, teledermatology, mobile phone, mobile applications, primary health care, smartphone, remote consultation

## Abstract

**Background:**

PhotoExam is a mobile app that incorporates digital photographs into the electronic health record (EHR) using iPhone operating system (iOS, Apple Inc)–based mobile devices.

**Objective:**

The aim of this study was to describe usage patterns of PhotoExam in primary care and to assess clinician-level factors that influence the use of the PhotoExam app for teledermatology (TD) purposes.

**Methods:**

Retrospective record review of primary care patients who had one or more photos taken with the PhotoExam app between February 16, 2015 to February 29, 2016 were reviewed for 30-day outcomes for rates of dermatology consult request, mode of dermatology consultation (curbside phone consult, eConsult, and in-person consult), specialty and training level of clinician using the app, performance of skin biopsy, and final pathological diagnosis (benign vs malignant).

**Results:**

During the study period, there were 1139 photo sessions on 1059 unique patients. Of the 1139 sessions, 395 (34.68%) sessions documented dermatologist input in the EHR via dermatology curbside consultation, eConsult, and in-person dermatology consult. Clinicians utilized curbside phone consults preferentially over eConsults for TD. By clinician type, nurse practitioners (NPs) and physician assistants (PAs) were more likely to utilize the PhotoExam for TD as compared with physicians. By specialty type, pediatric clinicians were more likely to utilize the PhotoExam for TD as compared with family medicine and internal medicine clinicians. A total of 108 (9.5%) photo sessions had a biopsy performed of the photographed site. Of these, 46 biopsies (42.6%) were performed by a primary care clinician, and 27 (25.0%) biopsies were interpreted as a malignancy. Of the 27 biopsies that revealed malignant findings, 6 (22%) had a TD consultation before biopsy, and 10 (37%) of these biopsies were obtained by primary care clinicians.

**Conclusions:**

Clinicians primarily used the PhotoExam for non-TD purposes. Nurse practitioners and PAs utilized the app for TD purposes more than physicians. Primary care clinicians requested curbside dermatology consults more frequently than dermatology eConsults.

## Introduction

The visual nature of skin conditions in dermatology has promoted the application of medical photography in the specialty. Technological advances in digital cameras, computer memory, and processing power have contributed to an expansion of potential applications of digital medical photography in dermatology practice. Digital photography facilitates communication with the health care team and patients and documentation for medical and academic purposes [1]. Multiple applications of digital photography in dermatology have emerged, including education, clinical archiving in patient records, surgical documentation pre- and postoperatively, follow-up of chronic conditions for progression or treatment response, diagnosis of melanocytic lesions, and teledermatology (TD) consultation [1].

TD is an established practice that enables remote access to dermatologic care using communications technology [2]. Three modes of TD care delivery are utilized: (1) store and forward (S&F) for transmitting digital images and clinical information to the dermatologist for consultation at a later time; (2) real-time video teleconferencing (VTC), where clinicians and patient interact live in videoconference; and (3) a hybrid model using a combination of the above. Store and forward represents the most commonly employed care delivery model for TD and may substitute for the dermatologic physical examination [3].

Uses of TD include consultation, triage, direct care for the diagnosis and management of skin disorders, and follow-up of chronic skin conditions [4]. Studies suggest that TD is cost-effective and associated with high levels of diagnostic accuracy, increased access to care, better clinical outcomes, and high levels of satisfaction reported by patients, referring clinicians, and dermatologists using this model [5-8]. TD via mobile phone apps has been targeted directly to consumers, as well as to clinicians [9]. Nelson et al demonstrated increased speed of dermatologic consultation and accessibility using S&F TD via a mobile app in an underserved primary care setting [10]. Nami et al found high levels of agreement between dermatologist in-person diagnoses and teledermatologist diagnoses when using asynchronous iPhone 4S (Apple Inc, Cupertino, CA, USA), skin images and clinical history from a mobile TD app (MugDerma e-derm-consult GmbH, Graz, Austria), [11]. A pilot study of resident physicians from emergency medicine, internal medicine, and dermatology that evaluated physician satisfaction with a mobile app for clinical photography found that the majority found the app useful and easy to use and desired to continue using the app [12]. Increasing access to mobile technologies with high quality cameras with or without electronic health record (EHR) integration, the rising burden of skin disease, uneven geographic distribution of dermatologists, prolonged wait times, and socioeconomic barriers to dermatologic care have together fueled the rise of TD consultative services across the United States [13]. Although privacy and confidentiality, image quality, and diagnostic confidence continue as barriers to widespread adoption [14-19], TD has great potential to minimize disparities in dermatologic care.

The purpose of this retrospective study was two-fold: (1) to describe usage patterns of a mobile iPhone operating system (iOS)–based app, PhotoExam, for clinical image capture of skin conditions in the primary care clinics of an integrated tertiary care system and (2) to assess clinician-level factors that influence the use of the PhotoExam app for TD purposes. Our primary outcome measures were 30-day post-S&F dermatology consultation request and mode of dermatology consultation (curbside consult, eConsult, and in-person consult). Our secondary outcome measures were specialty and training level of clinician using the app, performance of biopsy of skin condition of interest, and final pathological diagnosis (benign vs malignant).

## Methods

### Store and Forward Teledermatology Platform and Care Process

In 2015, Mayo Clinic launched PhotoExam, an internal app available to all clinicians that incorporates digital clinical photographs into the EHR using iOS-based mobile devices. In primary care practice, the promise with the app’s release was greater access to dermatologic expertise, with the ability to either speak with an on-call dermatologist via telephone for curbside consultation or submit a dermatology eConsult. Curbside consultations occur via telephone in a live interactive consultation between the primary care clinician and the dermatologist either with or without the patient present. For a curbside consultation, the referring clinician calls a dedicated pager that is carried by a dermatologist who is on call to respond to curbside consultations. During a curbside consultation, the clinical documentation of the discussion and recommendations are performed by the requesting primary care clinician. An eConsult is an asynchronous electronic free text-based consultation between the requesting clinician and the dermatologist, where medical information including the PhotoExam images, diagnostic tests, and specific questions are made available electronically to the dermatology specialist to enable timely access to dermatologic expertise. eConsults are ordered by the referring provider, and completion is tracked using the same electronic ordering system as in-person referrals. eConsults result in a formal dermatology note in the patient’s EHR.

PhotoExam is an app that can be downloaded by providers from an internal Mayo Clinic website to iPhone, iPad, or iPod touch devices when connected to the Mayo Clinic intranet. Once the clinician securely logs in to the patient record using the PhotoExam app, they are prompted to confirm that consent has been obtained for photography before accessing the photo capture screen. The provider is then prompted to enter the anatomical sites being photographed. Photos can then be taken in the app using the device’s camera. Clinical images are transferred to institutional imaging systems via a secure network, stored, and made available for viewing in the EHR within minutes. PhotoExam is available to be run on any iOS 7.2 or higher device that is connected to the institutional network. As the devices are limited to this platform, photo resolution is assured to at least equal the criteria in current TD practice guidelines (8 megapixel or greater) [6]. Because no patient data remains on the mobile device, clinicians can use their own personally owned devices to take photos; however, all devices using the app must have a security profile installed. No training in medical photography is provided or required before using the app. A previous review of system-wide use of the app (ie, not limited to the primary care setting or specifically to TD) revealed that the quality of photos taken with the app was judged as generally good, with images receiving, on average, 91% of possible points on the quality scoring rubric used for assessment [20].

### Study Design and Data Collection

We performed a retrospective review of the EHR for patients who had clinical digital images captured by primary care clinicians in family medicine, internal medicine, and general pediatrics using the PhotoExam app from February 16, 2015 to February 29, 2016. Patients who did not have research authorization on file were excluded from review. This study was approved by the institutional review board of the Mayo Clinic. The Mayo Clinic Advanced Cohort Explorer is a clinical data repository with text search functionality that was used to text search the EHR for the keywords “dermatology,” “dermatologic,” “dermatologist,” and “derm” and to search for pathology reports within 30 days after photo capture using the PhotoExam app. Medical records that contained the above search terms were then manually reviewed to assess for our primary outcomes. PhotoExam sessions represented our unit of study and were documented for each patient. A session was defined as all photos taken by a clinician of a single patient within a single calendar day. All data collection was performed by one coauthor (JLP).

Patient records were reviewed for the following outcomes within 30 days after photo capture: dermatology consultation, mode of dermatology consultation (curbside consult, eConsult, and/or in-person consult), performance of skin biopsy of the dermatologic condition captured with the PhotoExam app, medical specialty and training level of clinician using the app, and final pathologic diagnosis (malignant vs benign) of skin biopsy, if performed. For eConsults, we looked for statements from the responding dermatologist about the quality of the photographic images, noting particularly if the dermatologist made any comment on inadequacy of images for any reason. Descriptive statistics were used to summarize the data. Statistical analyses were performed using software, JMP Pro 12.0.0 (SAS Institute Inc). Excel 2010 (Microsoft) was used to generate a bubble chart of user types and specialties.

## Results

During the study period, 1139 discrete PhotoExam sessions were captured on 1059 unique patients. Table 1 outlines the characteristics of the cohort.

**Table 1:** Features of PhotoExam sessions.

Features		n (% or range), N=1059 patients (1139 photo sessions)
Age (years), mean		44 (0-104)
**Gender**		
	Female	564 (53.26%)
**Race**		
	White	917 (86.59%)
	Black	40 (3.78%)
	Asian	44 (4.15%)
	Other	36 (3.40%)
	Chose not to disclose	12 (1.13%)
	American Indian or Alaskan native	4 (<1%)
	Native Hawaiian or Pacific Islander	1 (<1%)
	Unknown	5 (<1%)
Photos per session, median		2 (1-18)
Number of body sites photographed per session, median		1 (1-6)
**Photo sessions by training level of clinician**		
	Consultant physician	567 (49.78%)
	Resident physician	155 (13.60%)
	NP^a^ and PA^b^	417 (36.61%)
**Photo sessions by medical specialty**		
	Internal medicine	589 (51.71%)
	Family medicine	282 (24.76%)
	Pediatrics	268 (23.53%)

^a^NP: nurse practitioner.

^b^PA: physician assistant.

**Figure 1:**
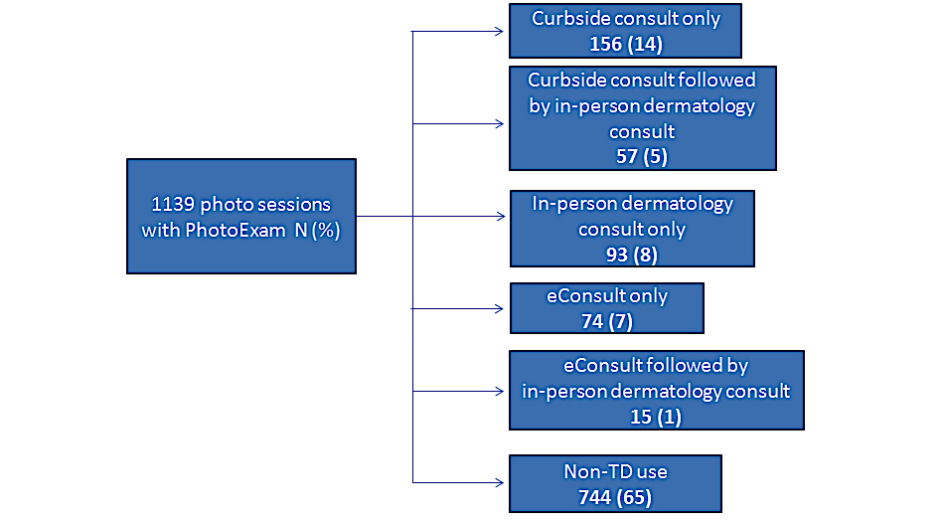
Flow chart of dermatology consultation rates and type of 1139 photo sessionsTD, Teledermatology.

Of the 1139 sessions, 395 (34.68%) sessions documented dermatologist input in the EHR via dermatology curbside consultation, eConsult, and in-person dermatology consult. Figure 1 demonstrates a flowchart showing patterns of dermatology consultation after use of the app by the requesting primary care clinician.

**Figure 2:**
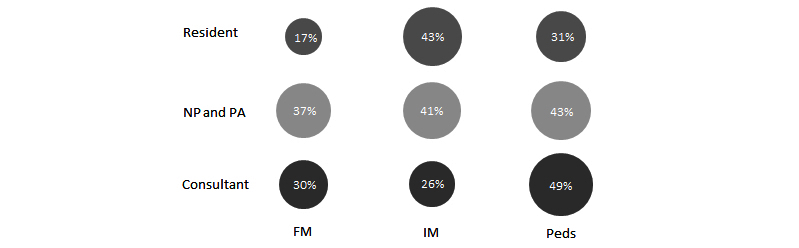
Bubble chart of percentage of photo sessions with dermatology input (via eConsult, curbside consult, and/or in person dermatology visit) by specialty and provider type. NP: nurse practitioner, PA: physician assistant, FM: family medicine, IM: internal medicine, PEDS: pediatrics.

The likelihood of requesting dermatology input on photos taken with the app varied significantly by both requesting clinician medical specialty and training level with NPs and PAs utilizing a higher proportion of photo sessions for TD consultation as compared with consultant and resident physicians (*P*=.03). Across the medical specialties in our cohort, pediatric clinicians utilized a higher percentage of photo sessions for dermatology consultation purposes as compared with family medicine and internal medicine clinicians (*P*=.003; Figure 2).

A total of 108 (9.5%) photo sessions had a biopsy performed of the photographed site, and only one biopsy was performed per session. Thus, 108 skin biopsies were performed; of these 46 biopsies (42.6%) were performed by a primary care clinician. Of the 108 photo sessions that had a biopsy of the site within 30 days of photo capture, 27 (25.0%) biopsies were interpreted as a malignancy (14 squamous cell carcinomas, 7 basal cell carcinomas, 3 melanomas, and 3 lymphomas). Of the 27 biopsies that revealed malignant findings, 6 (22%) had an eConsult or curbside consultation before biopsy, and 10 (37%) of these biopsies were obtained by primary care clinicians. Additionally, five dysplastic nevi were diagnosed on pathology, and four of these biopsies were obtained by primary care clinicians.

Review of the TD eConsults found that 16% (14/89) had statements from the responding dermatologist that suggested the images were not ideal for interpretation. In nine of these 14 statements, the comment from the dermatologist was that the photos were blurry or out of focus; however most commented that this was slight. For the other five, comments such as “difficult to ascertain” and “hard to tell” were used when describing the photos. An in-person dermatology visit was recommended by the dermatologist for nine out of the 14, whereas no in-person visit was recommended for the other five.

## Discussion

### Principal Findings

This study describes our early experiences with a mobile-based PhotoExam app for clinical image capture in the primary care setting. Our primary findings highlight usage of the PhotoExam app by primary care clinicians for multiple purposes including TD. Notably, the predominant use (approximately two-thirds of sessions) of the clinical images captured using the PhotoExam app in our cohort was for purposes other than TD.

Whereas the PhotoExam tool is most accurately characterized as supporting an S&F TD model of care, given its situation in mobile devices, it also allows for near real-time consultation on dermatologic concerns at or close to the point of care. The PhotoExam TD model is thus a hybrid that combines the advantages of efficient still clinical image capture as in S&F TD and the near real-time dialogue inherent to VTC. The seamless transfer of clinical images acquired using the PhotoExam app into the shared EHR enables ease of access and timeliness of consultation by dermatology specialists. This allows the primary care clinician to dermatologist consultation to occur by curbside (telephone) consult, eConsult, or in-person visit. With this app, primary care clinicians are able to choose between multiple pathways to dermatology advice, depending on the needs of the patient and clinician. When utilizing the PhotoExam app for TD, requesting clinicians utilized the curbside dermatology consultation option more frequently than the eConsult pathway. We observed that when the PhotoExam was used for TD curbside and eConsults, three-quarters of the time there was no in-person dermatology visit within the following 30 days, suggesting that PhotoExam app use, in conjunction with TD consultation, averted the need for an in-person dermatology visit in the majority of instances where used, a finding supported by a recent review of telehealth literature [21].

The finding that approximately two-thirds of these photo sessions by primary care clinicians did not have any evidence for dermatology consultation within 30 days suggests that this type of mobile technology has value to primary care clinicians beyond its use in TD. The limited literature supports various applications for digital clinical photography in dermatologic care, including education of medical students and physicians-in-training, enhancement of clinical documentation in practice, archiving patient records, pre- and postoperative documentation for referring physicians, and diagnosis of melanoma [1]. During our manual chart review, we saw various non-TD examples of how primary care clinicians were using this technology in practice. The most common reason appeared to be for simply documenting exam findings for descriptive purposes. However, we also saw examples where the stated intention was to document the physical examination to follow a condition over time (such as an evolving cellulitis or to follow acne after initiation of treatment). We saw instances in which the primary care clinician commented in their note that they obtained the photograph to be able to obtain TD input if their initial treatment of the skin disorder did not lead to resolution. When photographs were obtained before in-person dermatology consultations, we saw comments that photos were taken to document the finding on the day of the primary care visit, in case exam findings changed before the in-person dermatology consultation. We also saw examples of the app being used for documentation in nondermatologic settings, including hand injuries (used in conjunction with orthopedic and plastic surgery curbside consults), as well as documentation of ocular infection exam findings. Finally, we saw instances where clinicians used the app to capture photographs taken by patients on their own devices and shared with the clinician. We can envision myriad other possibilities for this app including presurgical or preprocedural consultations [22] and documenting acquired deformities, congenital malformations, and trauma findings [23,24].

NPs and PAs were more likely to utilize the PhotoExam for TD as compared with physicians, whereas pediatric clinicians were more likely to utilize the PhotoExam for TD as compared with family medicine and internal medicine clinicians. Though of a different methodological design, a study comparing eConsult referral patterns to specialists between NPs and physicians in family medicine found that NPs directed a higher proportion of their eConsults to dermatology when compared with family medicine physicians [25]. Nurse practitioners and PAs in our study had a fairly consistent use of the PhotoExam for TD (37%-43% of sessions) across specialties, whereas there was a much broader range for rates of physicians using PhotoExam for TD across specialties with a range of 17% to 49% of sessions utilizing TD among physicians. The difference in rates of using PhotoExam for TD across specialties appears to be driven by the very low rate of family medicine residents using the PhotoExam for TD (17% of sessions) and the high rate of pediatric staff physicians using the PhotoExam for TD (49% of sessions). Our study design does not allow us to assess whether this difference is because of some provider types requesting more TD services than other provider types (thus leading to a higher percentage of PhotoExam sessions for TD) or if the difference is because of some provider types utilizing the PhotoExam more frequently for non-TD purposes (such as clinical documentation). If the latter is the case, this could lead to a higher overall use of PhotoExam with a lower rate of using the PhotoExam for TD without representing a difference in overall TD rates between specialties or provider types per patients seen in clinic.

We found that the majority of photos submitted by primary care clinicians involved only one anatomic site, which suggests that primary care clinicians are either utilizing this primarily for discrete skin lesions or conditions rather than diffuse conditions or clinicians are only taking single site photos of diffuse conditions. We found only a very small percentage (2%) of the sessions were of lesions diagnosed as malignant, though it is possible that some of these lesions may have been diagnosed as malignant at a date after our data collection period.

Review of the times that the app was used for a TD eConsult revealed that lack of a clear photo was not a major driver for in-person dermatology exam recommendations as this was seen only 10% (9/89) of the time. We could not retrospectively examine what the curbside dermatologist thought about the quality of the photos submitted. Documentation of these curbside consultations was done by the primary care clinician rather than the responding dermatologist.

### Limitations

Limitations of our study include the retrospective nature of our review that limited our ability to ensure that the extracted data and the actions of the clinician were concordant. For example, clinicians may have performed a curbside consultation with a dermatologist but not documented that conversation in the EHR. We did not collect data on primary care TD consults before the release of the PhotoExam app, and therefore, we were unable to assess whether there was a change in these rates with implementation of the PhotoExam app. Data extraction was performed by only one reviewer, though a standard process was used. Though our review of eConsults suggested that poor image quality was not a major driver for in-person dermatology referrals, our study design did not allow us to determine how often this was a factor for in-person dermatology referrals after curbside consults. Finally, our study population was primarily white, which may limit generalizability.

### Conclusions

The PhotoExam app appears to be used as intended in primary care as a tool for TD. However, providers have also found other creative uses of the app, including augmenting textually constrained physical examination documentation, inputting into the medical record patient-taken photos (via a screenshot), documenting disease progression or regression, and consulting nondermatology specialists. Areas for future study include assessing health outcomes, app use in different medical settings, and clinician and patient satisfaction. We predict that the ubiquity of mobile devices with cameras and the availability of apps such as PhotoExam will accelerate the establishment of clinical photography as the standard of care for documentation in primary care and other areas of medicine.

## Abbreviations

EHR: electronic health record
iOS: iPhone operating system
NP: nurse practitioner
PA: physician assistant
S&F: store and forward
TD: teledermatology
VTC: video teleconferencing
